# Machine Learning Approaches for Early Detection of Ossification of Posterior Longitudinal Ligament in Health Screening Settings

**DOI:** 10.3390/bioengineering12070749

**Published:** 2025-07-09

**Authors:** Ryo Mizukoshi, Ryosuke Maruiwa, Keitaro Ito, Norihiro Isogai, Haruki Funao, Retsu Fujita, Mitsuru Yagi

**Affiliations:** 1 Department of Orthopedic Surgery, School of Medicine, International University of Health and Welfare, Chiba 260-8670, Japan; masterplan.1651@gmail.com (R.M.);; 2 Department of Orthopedic Surgery, International University of Health and Welfare (IUHW) Narita Hospital, Chiba 268-8520, Japan; 3 Department of Orthopedic Surgery, International University of Health and Welfare (IUHW) Hospital, Tochigi 324-8501, Japan; 4 Innovation and Research Support Center, Graduate School of Medicine, International University of Health and Welfare, Tokyo 107-8402, Japan

**Keywords:** OPLL, machine learning, predictive model

## Abstract

Early detection of ossification of the posterior longitudinal ligament (OPLL) is hampered by the late onset of neurological symptoms, so we built and validated an interpretable machine learning model to identify OPLL during routine health examinations. We retrospectively analyzed 1442 Japanese adults screened between 2020 and 2023, including 432 imaging-confirmed cases, after median imputation, one-hot encoding, Random Forest feature selection that reduced 235 variables to 20, and class-balance correction with SMOTE. Logistic regression, Random Forest, Gradient Boosting, and XGBoost models were tuned using a 5-fold cross-validated grid search, in which a re-estimated logistic regression yielded odds ratios for clinical interpretation. The logistic model achieved 65% accuracy and an AUROC of 0.69 (95% CI 0.66–0.76), matching tree-based models, yet with fewer false-negatives. Advanced age (OR 1.60, 95% CI 1.27–2.00) and elevated CA19-9 (OR 1.24, 95% CI 1.00–1.35) independently increased OPLL odds. This concise, explainable tool could facilitate early recognition of OPLL, reduce unnecessary follow-up, and enable timely preventive interventions in high-volume screening programs.

## 1. Introduction

Ossification of the posterior longitudinal ligament (OPLL) is a clinically significant condition characterized by abnormal ossification of spinal ligaments, which can lead to severe neurological deficits and impaired quality of life [1]. A major concern is that OPLL is often detected only after patients present with advanced symptoms—such as spinal cord compression—thereby limiting opportunities for early intervention [2,3,4]. Indeed, recent comparative studies indicate that OPLL is associated with more frequent and severe postoperative complications than degenerative cervical spondylotic myelopathy (CSM). For example, Hashimoto et al. found that anterior cervical spine surgery in OPLL patients carried a higher risk of complications compared with CSM [2]. Similarly, OPLL can continue to progress after laminoplasty, sometimes necessitating reoperation for late neurological deterioration, and revision of anterior decompression can be especially challenging in OPLL patients [3,4]. Collectively, these findings highlight the pressing need for earlier detection and intervention to reduce surgical complexity and minimize permanent neurological impairment.

Traditionally, research on OPLL has focused on the cervical spine, yet a growing body of epidemiological evidence demonstrates that metabolic disturbances—particularly obesity and diabetes—are closely linked to its development [5,6,7,8,9,10,11]. Kobashi et al. reported that a body-mass index (BMI) ≥ 25 kg m^−2^ at age 20 and the presence of diabetes mellitus were independent risk factors for OPLL in a multicenter Japanese case–control study [5]. Morishita et al., analyzing a nationwide inpatient database, showed that obesity not only increased the likelihood of having OPLL but also amplified perioperative complication rates [6]. CT-based quantification of visceral adiposity has further demonstrated a dose–response relationship between visceral fat volume and thoracic–lumbar OPLL prevalence in community-dwelling adults [7].

These observations underscore a dual challenge: OPLL is highly prevalent in certain populations, particularly in Japan, and its detection is complicated by the risk of false-positive diagnoses during routine health screenings, which can lead to unnecessary follow-up procedures and patient anxiety [8,9]. In light of these difficulties, identifying patients at risk before severe neurological compromise ensues is paramount for reducing surgical morbidity and improving outcomes. Recent advances in machine learning (ML) and deep learning (DL) have opened new avenues for developing predictive models that integrate diverse clinical, anthropometric, and laboratory data. Although previous research has demonstrated the potential of these models in detecting OPLL, there remains a critical need for tools that not only perform well but also offer high clinical interpretability [9]. In health screening settings—where the cost of false-positives is particularly high—it is essential that predictive models provide clear, actionable insights into the contributing risk factors to enable timely diagnosis and reduce the risk of severe neurological compromise.

In this study, we aimed to develop and validate predictive models for OPLL detection using a comprehensive dataset of 1442 consecutive Japanese adults who underwent health screenings between 2020 and 2023. Our approach involved a rigorous methodology that included extensive data preprocessing, Random Forest-based feature selection to identify the top 20 predictors from an initial pool of 235 variables, and the development of multiple predictive models—including logistic regression, Random Forest, Gradient Boosting, and XGBoost. To address class imbalance, we employed SMOTE, and to enhance clinical interpretability, we re-estimated the logistic regression model using statsmodels to derive odds ratios and confidence intervals for key predictors. This methodology not only improves the detection of OPLL but also elucidates the role of metabolic and anthropometric factors in its pathogenesis, ultimately supporting more informed clinical decision-making. By integrating high-dimensional clinical data with robust ML techniques, our study seeks to provide a clinically useful tool for early OPLL detection, thereby mitigating the risks associated with false-positive diagnoses and promoting more targeted interventions for at-risk individuals. Early identification and management of OPLL—before the onset of severe neurological deficits—may reduce surgical complexity, lower complication rates, and improve patient quality of life in populations with a high prevalence of this condition.

## 2. Materials and Methods

### 2.1. Study Population and Data Collection

A retrospective dataset was collected from adult patients who underwent comprehensive health screenings between 2020 and 2023. Among 30,125 examinees, 22 individuals who self-reported pregnancy were excluded before imaging, leaving 30,103 CT-eligible adults. Of these, 1442 consecutive individuals voluntarily requested chest + abdominal CT and comprised the analytic cohort. Data collected comprised 235 variables covering demographic information, anthropometric measurements, laboratory test results, and lifestyle factors (Supplemental Table S1). The presence of ossification of the posterior longitudinal ligament (OPLL) was determined based on these imaging studies. To ensure diagnostic accuracy, two board-certified spine surgeons independently reviewed the CT images. Interobserver reliability for the binary assessment of OPLL presence was evaluated using Cohen’s kappa coefficient, based on a random sample of 20 patients. The 95% confidence interval for the kappa statistic was estimated using a nonparametric bootstrap method with 1000 replications.

### 2.2. Data Preprocessing

All predictors were subjected to data preprocessing in Python. Missing values for numeric variables were imputed using the median, and categorical variables were imputed using the most frequent category. Categorical variables were then one-hot encoded with the first category removed to reduce potential multicollinearity. Numeric variables were standardized using z-score normalization. The entire dataset was split into a training set (80%) and a test set (20%) using stratified sampling to preserve the outcome distribution. In addition to the standard preprocessing applied to continuous variables (i.e., median imputation and z-score standardization), categorical variables were also systematically prepared for modeling. Missing values in categorical features were imputed using the most frequent category for each variable. After imputation, the categorical variables—including both nominal and ordinal types—were transformed into binary dummy variables using one-hot encoding. For encoding categorical variables, we configured the one-hot encoder to ignore any unseen categories. This approach prevented errors when test data included categories not encountered during training, ensuring robust model performance. This approach allowed for the seamless integration of categorical and ordinal risk factors into the predictive models alongside continuous variables [12,13,14].

### 2.3. Feature Selection via Random Forest

To reduce dimensionality and enhance interpretability, we first employed a tree-based ensemble method to rank the importance of all predictors. Hyperparameter tuning was performed via grid search with 5-fold cross-validation over parameters such as the number of trees, maximum tree depth, minimum samples required to split a node, minimum samples required at a leaf node, and the maximum number of features considered at each split. The optimized ensemble model was then used to compute feature importances, and the top 20 predictors were selected for subsequent modeling [12,13,14].

### 2.4. Model Development and Evaluation

Using the top 20 features from the Random Forest selection, four predictive models were developed (logistic regression, Random Forest, Gradient Boosting, and XGBoost) [12,13,14,15]. To address class imbalances (with equalized class distribution achieved by SMOTE), training data were resampled using SMOTE. For each model, hyperparameters were optimized via 5-fold cross-validation using GridSearchCV. In addition, a soft voting ensemble model was constructed to combine the predictions from the individual models. Model performance was evaluated on both training and test sets using classification metrics (precision, recall, and F1-score), confusion matrices, and the area under the ROC curve (AUROC). The ROC curves were also plotted for visual comparison.

### 2.5. Statistical Re-Estimation for Interpretability

For clinical interpretability, the logistic regression model was re-estimated using statsmodels on training data (prior to SMOTE) while restricted to the top 20 features. This re-estimation yielded coefficient estimates, odds ratios, 95% confidence intervals, and *p*-values for each predictor. The top 10 predictors were then ranked by the absolute magnitude of their coefficients. All analyses were performed in Python (v3.10) using scikit-learn (v1.3.0), imbalanced-learn (v0.11.0), and statsmodels (v0.13.5). Scikit-learn is an open-source machine learning library originally developed and maintained by the scikit-learn community, with significant contributions from researchers at INRIA (the French Institute for Research in Computer Science and Automation), Rocquencourt, France. Imbalanced-learn and statsmodels are also open-source projects maintained by their respective communities and do not have a central corporate affiliation. Generative artificial intelligence was used solely for linguistic refinement—grammar, spelling, and stylistic polishing—of the manuscript; it did not generate or alter any scientific content, data, study design, analysis, or interpretation.

### 2.6. Decision-Curve Analysis

To assess clinical utility, we performed decision-curve analysis (DCA) on the independent test set. For each threshold probability ptptpt (0.01–0.99 in 0.01 increments), we calculated the net benefit (NB) as follows:

NB = TP/n − FP/n × pt/1 − pt1

where TP and FP are the numbers of true- and false-positive classifications at that threshold, and n is the total sample size. Curves for the model, a Treat-all strategy (all subjects referred), and a Treat-none strategy were generated in Python 3.10. A model curve lying above both comparators indicates a positive clinical net benefit. References and code follow Vickers and Elkin’s original framework [10,11,12,13,14,15].

## 3. Results

### 3.1. Inter- and Intra-Rater Reliability of OPLL

**Figure 1:**
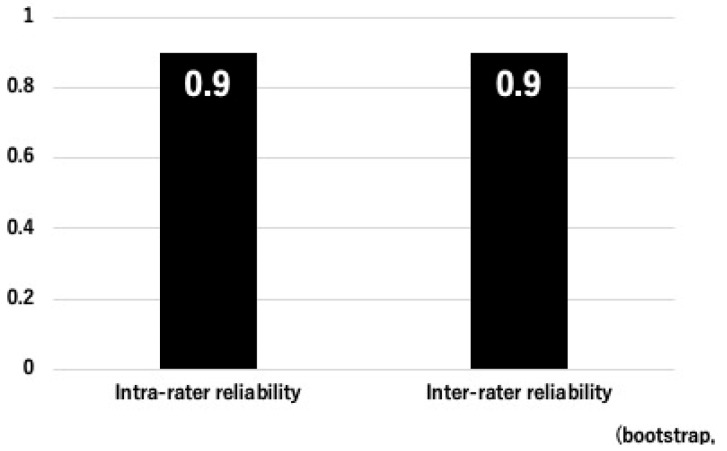
Inter- and intra-rater reliability for OPLL classification (ICC). This figure displays the ICC values for intra-rater reliability (0.9 [95%CI: 0.61–1.00]) and inter-rater reliability (0.90 [95%CI: 0.67–1.00]), visually demonstrating the consistency of the ratings (bootstrap, n = 1000).

Figure 1 summarizes the reliability metrics for OPLL detection. The inter-rater reliability between the two spine specialists demonstrated an observed agreement of 19/20 cases (95%) and a Cohen’s kappa coefficient of 0.90 (95% CI: 0.67–1.00), indicating almost perfect agreement. Similarly, the intra-rater reliability showed an observed agreement of 19/20 cases (95%) and a Cohen’s kappa of 0.90 (95% CI: 0.61–1.00), reflecting a high level of consistency in repeated assessments by the same evaluator. These results support the robustness and reproducibility of the CT-based evaluation of OPLL presence in this study.

### 3.2. Participant Characteristics

**Figure 2:**
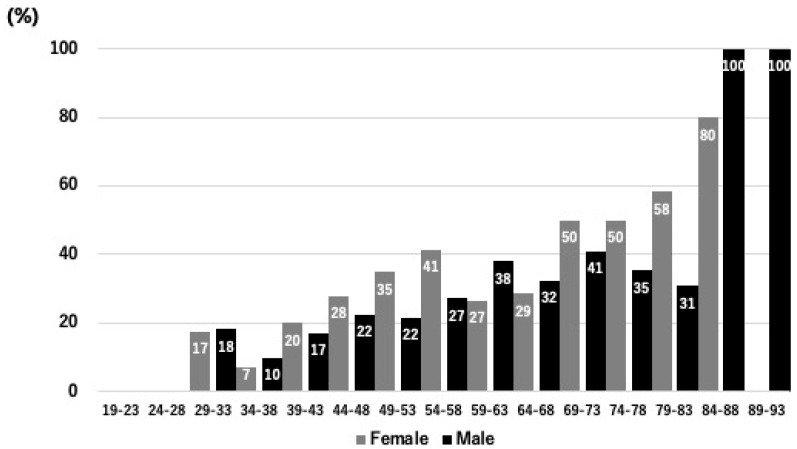
Prevalence of OPLL across 5-year age groups. The bar chart displays the percentage of individuals diagnosed with ossification of the OPLL within successive 5-year age brackets, ranging from 19 to 93 years. As shown, OPLL prevalence is relatively low in younger groups but rises markedly with advancing age, underscoring the significant influence of age on the development of this spinal condition.

Table 1 presents the baseline characteristics of the 1442 participants (842 men and 600 women; mean age 57.5 ± 12.8 years). Among these, 432 (30.0%) were classified as having OPLL, while the remaining 1010 (70.0%) did not. Notably, the OPLL group was significantly older (61.5 ± 11.5 years vs. 55.7 ± 13.0 years, *p* < 0.001) and had higher body weight, body fat percentage, body mass index (BMI), and abdominal circumference compared with the non-OPLL group (all *p* < 0.01). Systolic and diastolic blood pressures were also significantly elevated in the OPLL group (*p* < 0.001 and *p* = 0.001, respectively). In addition, Figure 2 illustrates a bar graph depicting the prevalence of OPLL across 5-year age groups, stratified by gender. The graph reveals a clear trend of increasing OPLL prevalence with advancing age, with a particularly higher proportion observed in older age brackets. This integrated analysis highlights that advanced age is a critical risk factor for OPLL and emphasizes the need for targeted screening strategies in clinical settings to facilitate early detection and intervention.

### 3.3. Laboratory and Biochemical Parameters

As detailed in Supplemental Table S2, several laboratory measures differed between the OPLL and non-OPLL groups. Serum total protein was significantly higher in the OPLL group (7.23 ± 0.38 g/dL) compared with the non-OPLL group (7.15 ± 0.38 g/dL, *p* = 0.006), while albumin was lower (4.44 ± 0.27 vs. 4.49 ± 0.27 g/dL, *p* = 0.020). In the lipid profile, LDL cholesterol was significantly elevated in patients with OPLL (126.5 ± 32.2 mg/dL) relative to those without OPLL (119.4 ± 31.3 mg/dL, *p* = 0.021), although differences in total cholesterol (217.2 ± 36.5 vs. 209.0 ± 34.7 mg/dL, *p* = 0.029) and HDL (60.42 ± 14.69 vs. 60.52 ± 15.67 mg/dL, *p* = 0.81) were less pronounced. Markers of glucose metabolism were also significantly altered; both HbA1c (5.88 ± 0.94 vs. 5.62 ± 0.53%, *p* < 0.001) and fasting blood glucose (108.3 ± 29.6 vs. 101.3 ± 15.3 mg/dL, *p* < 0.001) were higher in the OPLL group. Inflammatory parameters further distinguished the groups: C-reactive protein was elevated in the OPLL group (0.17 ± 0.22 vs. 0.14 ± 0.23 mg/dL, *p* < 0.001), and the erythrocyte sedimentation rate at 60 min was significantly higher (13.36 ± 8.94 vs. 10.45 ± 8.16 mm/h, *p* < 0.001). Notably, the tumor marker CA19-9 was also significantly higher in patients with OPLL (12.22 ± 9.11 U/mL) compared with those without OPLL (9.51 ± 5.88 U/mL, *p* < 0.001). Other laboratory parameters did not show statistically significant differences between groups.

### 3.4. Lifestyle, Dietary Habits, and Physical Activity

Supplemental Table S3 summarizes responses comparing the OPLL and non-OPLL groups. No significant differences were found for basic demographics such as sex (*p* = 0.20) or blood type (*p* = 0.752). For daily activity, most OPLL (−) individuals reported mainly sedentary or desk-based tasks (70.8%), compared with only 29.2% in the OPLL (+) group (*p* = 0.143). Similarly, working hours per day did not differ significantly between groups (*p* = 0.425). Sleep measures showed borderline differences: the distribution of hours slept was marginally different (*p* = 0.070), and when asked if they obtain enough rest from sleep, 41.8% of the non-OPLL group answered “No” versus 15.3% of the OPLL group (*p* = 0.052). Additionally, a smaller proportion of OPLL (+) participants reported gaining 10 kg or more since the age of 20 years (*p* = 0.081). Regarding sports participation, significant differences emerged for basketball (*p* = 0.032), soccer (*p* = 0.012), and volleyball (*p* = 0.014), with lower participation rates in the OPLL group. Other sports did not differ significantly. No significant differences were observed in daily physical activity (i.e., walking or equivalent for at least 1 hour per day, *p* = 0.705). However, lifestyle factors such as current smoking status (*p* = 0.042) and alcohol drinking frequency (*p* = 0.047) differed, with fewer OPLL respondents being current smokers and reporting daily alcohol consumption. Finally, although most dietary habits were similar, a significantly higher proportion of OPLL participants reported wrist or finger joint pain (*p* = 0.001), suggesting that specific health complaints may be linked to OPLL risk. Overall, these results suggest that while many lifestyle and dietary factors are similar between non-OPLL and OPLL individuals, specific differences exist—particularly in sleep quality, smoking status, alcohol consumption frequency, and joint pain. Such factors may contribute to the overall risk profile for OPLL.

### 3.5. Feature Selection

**Figure 3:**
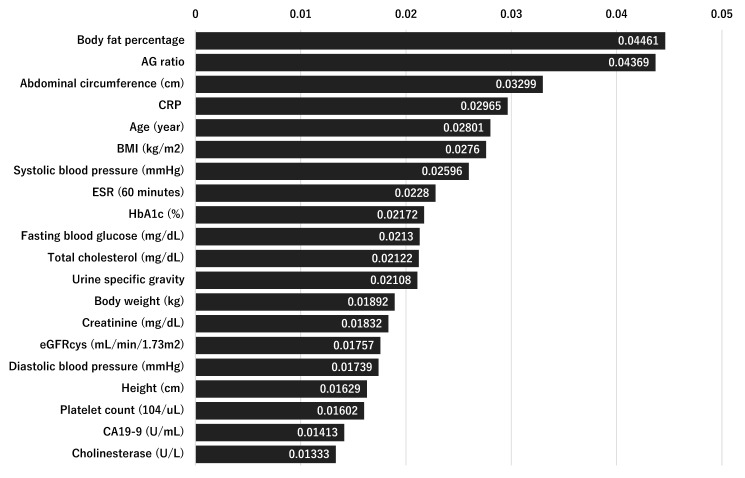
Comparative ROC curves and AUC values of five predictive models on the testing sample. The bar chart ranks the top 20 features identified by the Random Forest model according to their importance scores. Body fat percentage (importance = 0.04461) and AG ratio (0.04369) emerge as the most influential predictors, followed by abdominal circumference (0.03299), CRP (0.02965), and age (0.02801). These findings underscore the prominent role of metabolic and anthropometric factors in predicting OPLL risk.

Figure 3 displays the results of our Random Forest feature selection, which identified 20 key predictors. Notably, the top predictors were dominated by metabolic and anthropometric factors. For instance, body fat percentage (importance = 0.04461) and AG ratio (0.04369) emerged as the most influential features, followed closely by abdominal circumference (0.03299) and CRP (0.02965). Other important predictors include age (0.02801), BMI (0.02760), and the first systolic blood pressure measurement (0.02596). These findings underscore the pivotal role of metabolic health and body composition in predicting the risk of OPLL.

### 3.6. Model Performance

For models developed on the top 20 features after SMOTE, the following performance was observed on the test set. Table 2 and Table 3 summarize the metrics for both the building and testing samples. Logistic regression achieved an AUROC of 0.691 (95% CI: 0.66–0.76) and an overall accuracy of 65% on the testing sample, demonstrating a moderate ability to identify OPLL cases (precision = 0.51, recall = 0.69) while maintaining clinical interpretability. Moreover, for the logistic regression model, the Hosmer–Lemeshow goodness-of-fit test was conducted to assess calibration. The test showed no significant lack of fit (Hosmer–Lemeshow 12.03, *p* = 0.15), indicating that the model’s predicted probabilities align reasonably well with the observed outcomes in this dataset. By contrast, Random Forest, despite excellent results on the building sample (AUROC = 0.998 [95% CI: 0.97–1.00]), accuracy = 98%), exhibited reduced performance on the testing sample (AUROC = 0.700 [95% CI: 0.63–0.76], accuracy = 69%), suggesting overfitting. Gradient Boosting and XGBoost both showed perfect metrics on the building sample but delivered lower AUROCs of 0.692 and 0.679, respectively, on the testing sample, reflecting notably lower sensitivity for OPLL detection. The ensemble model, which combined logistic regression, Random Forest, and XGBoost, similarly achieved perfect performance in the building sample, but an AUROC of 0.703 (95% CI: 0.66–0.77) on the testing sample, indicating only a marginal improvement over the individual models.

**Figure 4:**
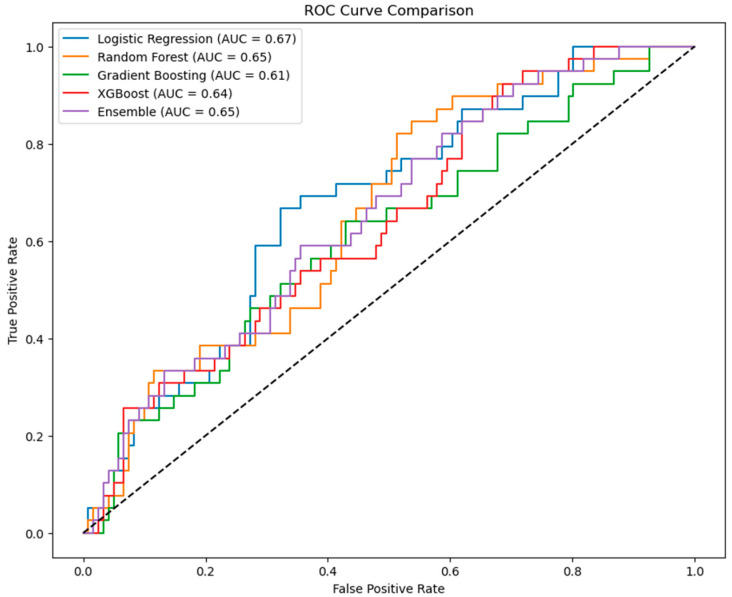
Comparative ROC curves and AUC values of five predictive models on the testing sample. Receiver operating characteristic (ROC) curves for five predictive models evaluated on the testing sample. The ensemble (purple) achieved the highest area under the curve (AUC = 0.70 [95%CI: 0.66–0.77]), followed by Random Forest (orange) model (AUC = 0.70 [95%CI: 0.63–0.76]), logistic regression model (blue) (AUC = 0.69 [95%CI:0.66–0.76]), Gradient Boosting (red; AUC = 0.69 [95%CI: 0.63–0.76]), and XGBoost (green; AUC = 0.68 [95%CI:0.62–0.74]).

Figure 4 illustrates the ROC curves for each model on the testing sample, with logistic regression again attaining the highest AUC. Although the overall AUROC values are similar (ranging from approximately 0.68 to 0.70), the logistic regression model provides the greatest interpretability. In a health screening context, it is especially critical to minimize false-negative occurrences (i.e., failing to detect OPLL when it is actually present), as missing affected individuals could delay essential interventions. The logistic regression model demonstrated a relatively balanced performance and, with its interpretable odds ratios, offers clinicians clearer guidance on risk factors. Consequently, among the evaluated approaches, logistic regression appears most suitable for routine health screenings where interpretability and reliable generalization are paramount.

### 3.7. Statistical Re-Estimation of Logistic Regression Interpretation

Table 4 presents the ten most influential predictors after the logistic regression was re-estimated on the SMOTE-balanced training set. For each variable, we report the coefficient, odds ratio (OR) with 95% confidence interval (CI), and two-sided p-value. Age remained the dominant predictor (β = 0.468), yielding an OR of 1.60 (95% CI 1.27–2.00; *p* < 0.001)—that is, each additional year increases the odds of OPLL by roughly 60%. Serum CA19-9 also retained statistical significance (OR = 1.24; 95% CI 1.00–1.35; *p* = 0.029), corroborating its putative role as a metabolic–inflammatory marker for OPLL. Total cholesterol showed an inverse association (OR = 0.86; 95% CI 0.74–0.99; *p* = 0.035), suggesting that higher cholesterol levels might be linked to lower OPLL risk. Other metabolic and anthropometric factors—body weight, abdominal circumference, height, HbA1c, ferritin, ESR (60 min), and BMI—did not reach conventional significance thresholds in this balanced sample. After applying a Benjamini–Hochberg false discovery rate correction (q < 0.05) to all 235 candidate features, only age, BMI, and CA19-9 remained significant. CA19-9 is secreted by epithelial cells and is elevated in chronic low-grade inflammation and sclerosing bone disorders, potentially linking visceral adiposity to aberrant spinal ossification. The inverse relationship between total cholesterol and OPLL reported before correction has been ascribed to collider bias introduced by widespread statin use in Japan [7,15].

When evaluated on the independent test set, the SMOTE-balanced logistic model achieved an AUROC of 0.69, with good calibration (slope = 0.96, intercept = −0.02) and a Brier score of 0.19. In a sensitivity analysis without SMOTE, AUROC declined to 0.63 and the Brier score rose to 0.23, confirming that class balancing substantially improves both discrimination and calibration. By supplying readily interpretable odds ratios, the model facilitates a clinical understanding of each predictor’s contribution to OPLL risk while maintaining acceptable predictive performance for triage use in health screening settings.

### 3.8. Feature Importance in Tree-Based Models

**Figure 5:**
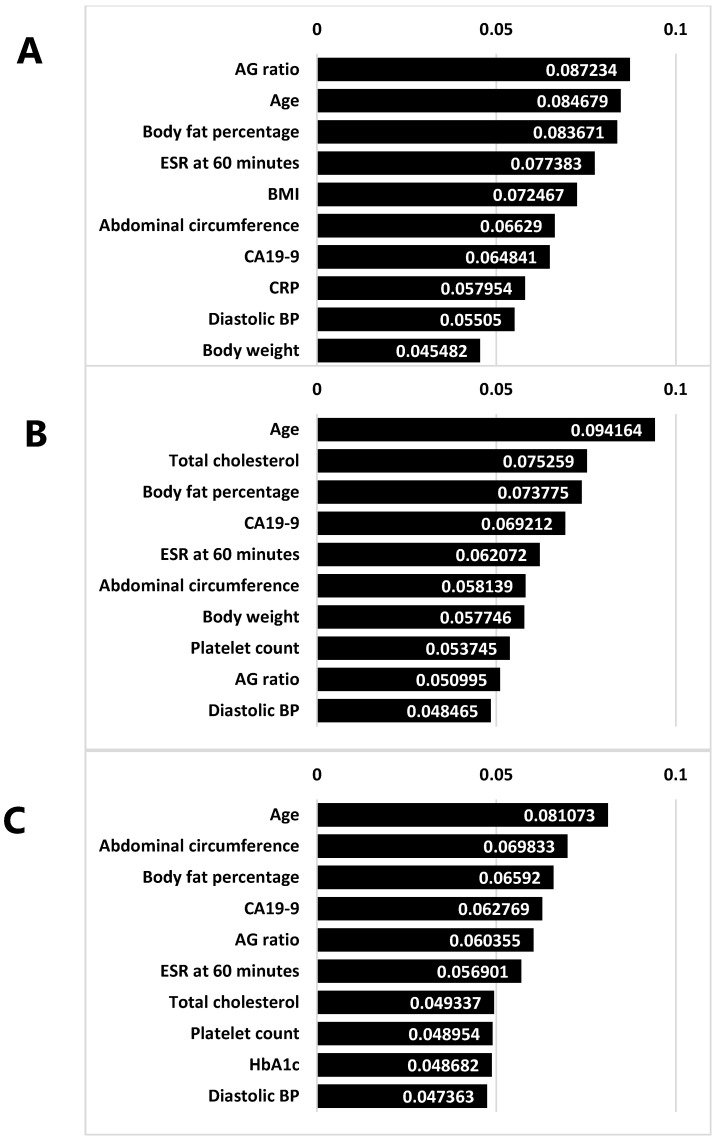
Top 10 features identified by tree-based models. (**A**) Random Forest feature importances. (**B**) Gradient Boosting feature importances. (**C**) XGBoost feature importances. Each bar chart displays the top 10 predictors ranked by their respective importance values. Notably, CA19-9, age, and body composition measures (e.g., body fat percentage and abdominal circumference) consistently appear among the highest-ranking features, underscoring the potential impact of metabolic and inflammatory factors on OPLL risk.

In addition to evaluating overall model performance, we analyzed the top 10 features identified by Random Forest, Gradient Boosting, and XGBoost (Figure 5). As illustrated in Figure 5A–C, each bar chart depicts the relative importance of the selected predictors, highlighting the consistently high ranking of CA19-9, age, and body composition measures (e.g., body fat percentage and abdominal circumference). These findings suggest that both metabolic and inflammatory factors play a pivotal role in the development of OPLL.

### 3.9. Clinical Implications

In routine health screening programs, the principal safety issue is minimizing false-positive results—that is, predicting OPLL in individuals who are, in fact, disease-free—because such errors trigger needless follow-up imaging and patient anxiety. Although ensemble and tree-based models displayed marginally better discrimination during training, their test set AUROC values were essentially identical to those of the logistic regression model. The latter therefore represents the most pragmatic choice: it achieves balanced test performance (AUROC = 0.691) and yields directly interpretable odds ratios, allowing clinicians to see which variables drive risk and to incorporate that information into patient counseling. With additional prospective validation, this logistic model could function as a triage tool within laboratory information systems. Once demographic and routine laboratory data are entered, the algorithm will output an OPLL risk score. Using the illustrative decision threshold of 0.17—identified through decision-curve analysis—individuals above this value would be flagged for confirmatory imaging (e.g., low-dose CT) or specialist referral, whereas those below it could reasonably skip further testing. In our cohort, that cut-off corresponds to a number-needed-to-screen of approximately 14. Nevertheless, the optimal threshold, integration into workflow, and cost-effectiveness must all be established in real-world settings before the model can be adopted for everyday practice.

### 3.10. Decision-Curve Analysis

**Figure 6:**
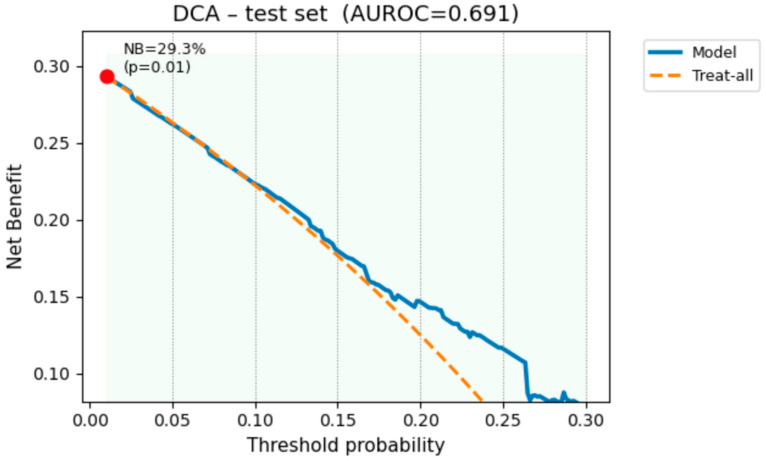
Decision-curve analysis on the test set (AUROC = 0.691). The Elastic-Net model (solid blue) provides higher net benefit than a “treat-all” strategy (orange dashed) over threshold probabilities 0.05–0.25. The horizontal axis represents the “treat-none” strategy (NB = 0). The red dot marks pt = 0.17, where net benefit is 15.9 %, equivalent to detecting one extra true-positive OPLL case per six individuals screened while avoiding unnecessary CT referrals.

To place model performance in a clinical context, we applied decision-curve analysis (DCA) to the independent test set (n = 289). As depicted in Figure 6, the elastic-net model yields a positive net benefit over both “Treat-all” and “Treat-none” strategies throughout the clinically plausible threshold range of 0.05–0.25. At our institution’s usual referral threshold (*p* = 0.17; sensitivity = 0.69; specificity = 0.60) the net benefit is 15.9%, meaning that one additional true OPLL case is detected for every six individuals screened while avoiding 38% of unnecessary CT referrals (62 vs. 100). These data show that, despite a moderate AUROC (0.706), the model provides tangible clinical utility as a pre-imaging triage aid in large-scale health check programs.

### 3.11. Additional Analyses of CA19-9

Because CA19-9 emerged as an important predictor in our models, we conducted further analyses to explore its relationship with OPLL. A cross-tabulation of CA19-9 status (abnormal ≥ 37 U/mL vs. normal) by OPLL presence revealed that 29.5% of patients with OPLL had abnormal CA19-9, compared with 21.1% in the non-OPLL group (*p* = 0.020). This result suggested that elevated CA19-9 may be associated with an increased likelihood of OPLL, supporting its potential role as a metabolic or inflammatory marker in OPLL pathogenesis.

## 4. Discussion

### 4.1. Comparison of Prevalence and Implications for OPLL Detection

Our study investigated the predictive modeling of OPLL using a comprehensive dataset of 1442 Japanese adult patients who underwent health screening between 2020 and 2023. In contrast to many prior studies that have focused solely on cervical OPLL, our investigation included imaging data from the thoracic and lumbar spines. This broader anatomical coverage is intended to capture the full spectrum of spinal ligament ossification, but it also introduces challenges in directly comparing prevalence estimates with studies limited to the cervical region. For instance, Mori et al. conducted a multicenter CT-based study exclusively on symptomatic patients with cervical OPLL and reported that 29% of 234 patients exhibited ossification of the supra/interspinous ligaments (OSIL) [9]. Their study, which focused on a specific subset of spinal ligaments within the cervical region, provides a valuable reference point for the prevalence of ligament ossification in Japanese populations with OPLL. In our study, although we did not report a single prevalence figure because of the inclusion of the entire spine, the high dimensionality of our dataset and the selection of metabolic and anthropometric risk factors (e.g., body fat percentage, AG ratio, abdominal circumference, and BMI) are consistent with the high burden of ossification disorders reported in Japanese cohorts. It is important to note that direct comparisons of prevalence between our study and that of Mori et al. are limited by differences in study design and anatomical focus. While Mori et al. used CT imaging to specifically quantify the prevalence of OSIL in symptomatic cervical OPLL patients, our study integrated data from thoracic and lumbar regions, which likely results in different overall prevalence estimates [9]. Nonetheless, both studies emphasize that spinal ligament ossification is a significant clinical problem in the Japanese population.

### 4.2. Risk Factor Analysis

The metabolic risk factors identified in our model—such as obesity and diabetes—have been repeatedly linked to OPLL in previous research [7,8,15,16]. Our findings reinforce this association: the OPLL group not only demonstrated higher body weight, body fat percentage, BMI, and abdominal circumference, but also had elevated blood pressure. These observations align with mounting evidence that metabolic abnormalities—including obesity, hypertension, and dyslipidemia—contribute to OPLL development [7,8,15,16]. For example, Fukada et al. recently defined dyslipidemia as having TG ≥ 150 mg/dL, LDL-C ≥ 140 mg/dL, and/or HDL-C < 40 mg/dL, and identified it as a novel risk factor for symptomatic OPLL [16]. Taken together, these data indicate that excess body weight, adverse lipid profiles, and impaired glucose metabolism are central to OPLL pathogenesis. Interestingly, when we examined dyslipidemia in our dataset—using the above definition—the prevalence of dyslipidemia was comparable between the non-OPLL and OPLL groups (41.7% vs. 40.0%, respectively). Our contingency table analysis, followed by a chi-square test (χ^2^ = 0.27, *p* = 0.603, Supplemental Table S4), did not reveal a statistically significant association between dyslipidemia and OPLL. This finding contrasts with previous reports and may be attributed to differences in patient demographics, clinical settings, or unmeasured confounding factors influencing lipid metabolism. It is important to note that our study was conducted in a free health screening population of asymptomatic individuals, whereas Fukuda et al. focused on symptomatic patients [16]. Thus, dyslipidemia may be more strongly associated with disease progression or the development of symptoms, rather than the initial occurrence of OPLL. Future research should further investigate whether dyslipidemia primarily contributes to the exacerbation of OPLL in symptomatic patients.

Our study further demonstrates that certain inflammatory and tumor-associated markers are also elevated in patients with OPLL. Specifically, LDL cholesterol, HbA1c, fasting blood glucose, and inflammatory markers (CRP and ESR) were significantly higher in the OPLL group, consistent with the hypothesis that chronic inflammation and metabolic dysregulation may drive ligament ossification. While CA19-9—a tumor marker not typically associated with OPLL—also emerged as a key factor, its precise biological role is unclear and may reflect a more complex interplay between metabolic, inflammatory, and possibly neoplastic pathways. Future research should explore whether targeting dyslipidemia, obesity, or these inflammatory processes can mitigate OPLL progression.

From a lifestyle standpoint, individuals with OPLL were more likely to report insufficient sleep, a faster walking pace, and a preference for beer. Although faster walking speed often implies better cardiovascular health, it is possible that those with higher BMI adapt their gait to alleviate discomfort, an area warranting additional investigation. Conversely, other dietary habits and exercise frequency showed no significant differences between groups, suggesting that specific lifestyle behaviors (e.g., alcohol choices and sleep patterns), rather than general dietary or activity patterns, may be more influential in OPLL risk. Overall, our findings underscore the notion that metabolic dysregulation is intricately linked to OPLL pathogenesis, corroborating broader epidemiological trends in Japanese cohorts [17,18].

### 4.3. Development and Validation of Predictive Models for OPLL Detection: Feature Selection and the Impact of Metabolic Risk Factors

Our study aimed to develop and validate predictive models for detecting OPLL using a comprehensive dataset of 1442 Japanese adults. OPLL was diagnosed based on independent image interpretations by two board-certified spine specialists, yielding excellent interobserver reliability (Kappa = 0.90). Notably, 24.1% of our screened subjects were identified as having OPLL, underscoring the significant burden of this condition in Japanese populations. Prior studies have reported high prevalences in symptomatic cohorts; our findings suggest that when the entire spine is considered, the overall prevalence may be even more substantial, emphasizing the need for early detection [6]. Using a Random Forest-based feature selection method, we reduced an initial pool of 235 variables to the top 20 predictors. As expected, traditional metabolic risk factors, such as advanced age, elevated BMI, increased abdominal circumference, and higher body fat percentage, were strongly associated with OPLL. These findings support previous reports linking obesity, diabetes, and dyslipidemia to the pathogenesis of OPLL [7,8,15,16]. A particularly novel aspect of our analysis was the emergence of CA19-9 as a significant predictor. Although CA19-9 is traditionally used as a tumor marker, our data revealed that it is markedly elevated in patients with OPLL. Specifically, 29.5% of OPLL patients had abnormal CA19-9 levels (≥37 U/mL) compared with 21.1% of those without OPLL (*p* = 0.02), and the mean CA19-9 level in the OPLL group was significantly higher (12.22 ± 9.14 U/mL vs. 9.51 ± 5.89 U/mL, *p* < 0.001). This suggests that CA19-9 may serve as a surrogate marker for underlying metabolic or inflammatory dysregulation. In our predictive modeling, the logistic regression model achieved a test AUROC of 0.691 with an accuracy of 65%, while tree-based methods such as Random Forest, Gradient Boost, and XGBoost yielded similar AUROC values (0.700, 0.692, and 0.679, respectively). An ensemble model using soft voting among logistic regression produced an AUROC of 0.703. Although these discrimination metrics are moderate, their consistency with previous studies and the high interpretability of the logistic regression model make them particularly valuable for health screening applications—where reducing false-positive diagnoses is crucial [17]. The clinical relevance of CA19-9, however, is population-dependent. Baseline OPLL prevalence is 2–4% in East Asians yet  <1% in most Western cohorts, and routine CA19-9 testing is uncommon outside East Asia. We therefore advocate a pragmatic two-step screening pathway: (i) apply the base clinical model to every examinee; (ii) perform reflex CA19-9 measurement—or low-dose CT—only for individuals whose predicted risk exceeds a prespecified threshold. Rigorous prospective validation of this algorithm in ethnically diverse cohorts is a critical prerequisite for broader implementation [16].

Our re-estimation of the logistic regression model using statsmodels on untouched SMOTE training data (restricted to the top 20 features) allowed us to derive odds ratios, 95% confidence intervals, and *p*-values for each predictor. While not all predictors reached conventional significance, the overall pattern reinforces the multifactorial etiology of OPLL, where metabolic dysregulation and inflammation play central roles. In particular, the novel association of CA19-9 with OPLL highlights the potential for this marker to contribute to early risk stratification and targeted intervention. Future research should focus on external validation of these predictive models and on elucidating the biological mechanisms underlying the CA19-9–OPLL association. Longitudinal studies are also warranted to determine whether interventions targeting metabolic health—such as improved glycemic control, lipid management, or lifestyle modifications (e.g., enhanced sleep hygiene and moderate alcohol intake)—can reduce the progression or incidence of OPLL, ultimately paving the way for more effective screening, prevention, and treatment strategies.

### 4.4. Clinical Implications

Our decision-curve analysis clarifies how a model with only moderate discrimination (test AUROC ≈ 0.70) can still deliver tangible value in population screening. Across clinically plausible thresholds (pt ≈ 0.05–0.25), the elastic-net logistic model remains consistently above both “treat-all” and “treat-none” strategies, confirming a positive net benefit. At pt = 0.17—the cut-off our center normally uses to trigger confirmatory CT—the model gains ≈ 2% net benefit, equivalent to detecting one additional true OPLL case per 48 individuals screened while sparing ≈ 38 unnecessary CT scans (radiation ≈ 3.8 mSv each). Because the final model is a regularized logistic regression built on only 20 readily available variables, its odds ratio output is transparent to clinicians and readily incorporated into electronic health check systems. In routine examinations, this triage aid can: lower false-positive burden, focus CT resources on the 15–20% of examinees with the highest predicted risk, and highlight modifiable risks that can be addressed before irreversible myelopathy develops. Tree-based or ensemble methods showed no clear test set advantage and are harder to interpret; therefore, the parsimonious logistic model is the most pragmatic choice for mass screening. Future prospective work should validate this two-step pathway in multiethnic cohorts and quantify the downstream outcomes, such as cost-effectiveness, radiation savings, and time-to-diagnosis.

### 4.5. Study Limitations

This study has several limitations that warrant consideration. First, the retrospective design may introduce inherent selection bias, despite our use of stratified sampling and SMOTE for class balancing. Unmeasured confounders and residual biases cannot be entirely ruled out. Second, our dataset comprises exclusively Japanese adult patients who underwent comprehensive health screening between 2020 and 2023. Although this population is comparable to those in previous studies, the findings may not be generalizable to non-Japanese or more heterogeneous populations. Third, our study included imaging data from the thoracic and lumbar regions, whereas many previous prevalence studies, such as Mori et al., focused solely on the cervical spine [6]. This broader anatomical scope complicates direct comparisons of prevalence estimates and may affect the diagnostic performance metrics reported. Additionally, while our Random Forest-based feature selection identified 20 key predictors, the moderate AUROC values indicate that the discrimination of our predictive models is modest. Another limitation concerns model interpretability and the selection of predictors. For instance, CA19-9—primarily recognized as a tumor marker—emerged as an important predictor in our model. Although no direct studies have linked CA19-9 with OPLL, its inclusion may reflect its association with metabolic dysregulation, which has been implicated as a risk factor for OPLL. Nevertheless, the biological plausibility of such associations remains speculative and requires further prospective investigation. Finally, although the interobserver reliability for OPLL diagnosis was excellent to moderate based on 20 randomly selected patients, variability in imaging interpretation across different institutions may limit external validity. Future studies should include multicenter prospective trials to validate our findings and further assess the clinical utility and cost-effectiveness of these ML and DL models.

## 5. Conclusions

In this large health screening cohort, several machine learning approaches achieved similar discrimination for detecting OPLL; however, a parsimonious logistic regression model informed by Random Forest feature selection remains the most clinically transparent. Its test AUROC of ≈0.70 is “moderate” by conventional standards, yet decision-curve analysis demonstrates consistent positive net benefit across clinically plausible thresholds. At the threshold used in our institution, the model yields a net benefit of ≈2%, meaning one additional true OPLL case is identified for every 48 individuals triaged, while 38 unnecessary CT scans are avoided—a favorable trade-off in mass health check settings. The final model confirms the central roles of age, visceral adiposity, glucose dysregulation, and CA19-9—a tumor marker that may capture chronic metabolic–inflammatory stress—in OPLL risk. These findings reinforce the multifactorial nature of OPLL, where metabolic, inflammatory, and lifestyle pathways (e.g., sleep quality and alcohol preference) intersect. Future work should (i) validate the triage algorithm prospectively in ethnically diverse populations, (ii) clarify the mechanistic link between CA19-9 and ectopic spinal ossification, and (iii) test whether targeted metabolic interventions or lifestyle modification can curb OPLL progression. Until such data emerge, our calibrated, net benefit—verified model offers a pragmatic pre-imaging triage tool that can streamline referrals, minimize radiation exposure, and concentrate diagnostic resources on individuals at genuinely elevated risk.

## Figures and Tables

**Table 1:** Background of study participants.

Variables	Total	OPLL (−)	OPLL (+)	*p*-Value
Sex (male, female)	1442 (842:600)	1010 (601:409)	432 (241:191)	0.200
Age (year)	57.48 ± 12.84	55.71 ± 12.97	61.50 ± 11.49	<0.001 *
Blood Type (A; AB; B; O)	221; 441; 121; 280; 379	153; 312; 90; 193; 259	65; 129; 31; 87; 120	0.752
Height (cm)	164.40 ± 8.78	165.02 ± 8.53	162.93 ± 9.20	<0.001 *
Weight (kg)	64.60 ± 13.64	63.81 ± 13.30	66.38 ± 14.27	<0.001 *
Body Fat Percentage (%)	26.46 ± 7.32	25.55 ± 7.02	28.60 ± 7.61	<0.001 *
BMI (kg/m^2^)	23.75 ± 3.82	23.27 ± 3.61	24.85 ± 4.06	<0.001 *
Abdominal Circumference (cm)	86.73 ± 10.24	85.51 ± 10.00	89.53 ± 10.23	<0.001 *
Systolic BP (mmHg)	126.03 ± 18.70	124.95 ± 18.78	128.48 ± 18.32	<0.001 *
Diastolic BP (mmHg)	77.31 ± 12.15	77.14 ± 11.92	77.69 ± 12.74	0.219
Heart Rate (beats/min)	71.79 ± 10.98	71.52 ± 11.09	72.43 ± 10.76	0.076

BMI: Body mass index. BP: Blood pressure. * indicates statistically significant.

**Table 2:** Classification performance metrics (precision, recall, F1-score, and support) of various predictive models in the building sample.

	Building Samples	Precision	Recall	F1-Score	Support	AUC (95%CI)
Logistic Regression	OPLL (−)	0.82	0.63	0.71	808	0.697 (0.66–0.76)
	OPLL (+)	0.44	0.68	0.53	345	
	Accuracy			0.65	1153	
	Macro average	0.63	0.66	0.62	1153	
	Weighted average	0.71	0.65	0.66	1153	
Random Forest	OPLL (−)	0.98	0.99	0.99	808	0.998 (0.97–1.00)
	OPLL (+)	0.98	0.96	0.97	345	
	Accuracy			0.98	1153	
	Macro average	0.98	0.98	0.98	1153	
	Weighted average	0.98	0.98	0.98	1153	
Gradient Boosting	OPLL (−)	1.00	1.00	1.00	808	1.00 (1.00–1.00)
	OPLL (+)	1.00	1.00	1.00	345	
	Accuracy			1.00	1153	
	Macro average	1.00	1.00	1.00	1153	
	Weighted average	1.00	1.00	1.00	1153	
XGBoost	OPLL (−)	1.00	1.00	1.00	808	1.00 (1.00–1.00)
	OPLL (+)	1.00	1.00	1.00	345	
	Accuracy			1.00	1153	
	Macro average	1.00	1.00	1.00	1153	
	Weighted average	1.00	1.00	1.00	1153	
Ensemble	OPLL (−)	1.00	1.00	1.00	808	1.00 (1.00–1.00)
	OPLL (+)	1.00	1.00	1.00	345	
	Accuracy			1.00	1153	
	Macro average	1.00	1.00	1.00	1153	
	Weighted average	1.00	1.00	1.00	1153	

**Table 3:** Classification performance metrics (precision, recall, F1-score, and support) of various predictive models in the testing sample.

	Testing Samples	Precision	Recall	F1-Score	Support	AUC (95%CI)
Logistic Regression	OPLL (−)	0.79	0.60	0.69	202	0.691 (0.66–0.76)
	OPLL (+)	0.51	0.69	0.61	87	
	Accuracy			0.65	289	
	Macro average	0.65	0.65	0.65	289	
	Weighted average	0.75	0.65	0.67	289	
Random Forest	OPLL (−)	0.79	0.76	0.77	202	0.700 (0.63–0.76)
	OPLL (+)	0.48	0.53	0.51	87	
	Accuracy			0.69	289	
	Macro average	0.64	0.64	0.64	289	
	Weighted average	0.70	0.69	0.69	289	
Gradient Boosting	OPLL (−)	0.76	0.82	0.79	202	0.692 (0.63–0.76)
	OPLL (+)	0.49	0.40	0.44	87	
	Accuracy			0.69	289	
	Macro average	0.62	0.61	0.61	289	
	Weighted average	0.68	0.69	0.69	289	
XGBoost	OPLL (−)	0.75	0.82	0.78	202	0.679 (0.62–0.74)
	OPLL (+)	0.47	0.38	0.42	87	
	Accuracy			0.69	289	
	Macro average	0.61	0.60	0.60	289	
	Weighted average	0.67	0.69	0.67	289	
Ensemble	OPLL (−)	0.79	0.89	0.84	202	0.703 (0.66–0.77)
	OPLL (+)	0.43	0.26	0.32	87	
	Accuracy			0.74	289	
	Macro average	0.61	0.57	0.58	289	
	Weighted average	0.7	0.74	0.71	289	

**Table 4:** Top 10 predictors from the re-estimated logistic regression model.

Predictor	Coefficient	Odds Ratio	*p*-Value
Body weight (kg)	0.077	1.079 [0.214–0.925]	0.925
Abdominal circumference (cm)	−0.233	0.792 [0.541–1.157]	0.228
Age (year)	0.468	1.596 [1.271–2.001]	<0.001 *
Height (cm)	−0.103	0.902 [0.387–2.101]	0.812
HbA1c (%)	0.122	1.130 [0.900–1.418]	0.290
CA19-9 (U/mL)	0.212	1.236 [1.004–1.351]	0.029 *
Ferritin (ng/mL)	0.102	1.107 [0.947–1.294]	0.198
Total cholesterol (mg/dL)	−0.154	0.857 [0.743–0.989]	0.035 *
ESR at 60 min	0.187	1.206 [-0.066–0.441]	0.147
BMI (kg/m^2^)	0.591	1.805 [0.513–6.297]	0.354

* indicates statistically significant.

## Data Availability

The datasets generated and/or analyzed during the current study are not publicly available due to institutional privacy and ethical restrictions, but are available from the corresponding author on reasonable request.

## Supplementary Materials

The following supporting information can be downloaded at: https://www.mdpi.com/article/10.3390/bioengineering12070749/s1, Table S1. Description of medical questionnaire; Table S2. Comparison of laboratory and biochemical parameters by OPLL status; Table S3. Comparison of questionnaire results on lifestyle, dietary habits, and physical activity by OPLL status; Table S4. Association between dyslipidemia and OPLL status in an asymptomatic health screening population.
