# Supplementary material for: Machine Learning Approaches for Early Detection of Ossification of Posterior Longitudinal Ligament in Health Screening Settings
Source: Bioengineering (Basel). 2025 Jul 9;12(7):749. doi: 10.3390/bioengineering12070749 (PMC12292339; doi:10.3390/bioengineering12070749)
Supplement: Supplementary file 1 [file bioengineering-12-00749-s001.zip › bioengineering-3721386-supplementary.pdf]

SUPPLEMENTAL TABLES

Table S1. Description of Medical Questionnaire

<u>Physical Activity and Exercise Habits</u><br>How active are you on a daily basis?<br>· Mostly sitting, with light activity like walking or shopping for about an hour per day.<br>· Frequently standing for tasks, like housework or work that involves walking or standing for up to 2 hours.<br>· Daily physical activity for about an hour, like walking or cycling, with some heavier tasks like farming or fishing.<br>· Mostly doing physical labor, such as construction work, for about 1 hour.<br>· Heavy manual labor for about an hour a day.<br>How many hours do you work or engage in activities per day?<br>· Less than 3 hours<br>· 3 to 6 hours<br>· 6 to 9 hours<br>· 9 to 12 hours<br>· 12 to 15 hours<br>· More than 15 hours<br>What type of work schedule do you follow?<br>· Day shift only<br>· Shift work<br>· Night shift only<br>· Other:<br>How many hours of sleep do you get?<br>· Less than 5 hours<br>· 5 to 7 hours<br>· 7 to 9 hours<br>· More than 9 hours<br>Do you sleep well?<br>· Yes, I sleep well.<br>· I often wake up in the middle of the night.<br>· I have shallow sleep.

· I wake up early.

· I currently take sleeping pills.

Are you getting enough rest from your sleep?

Yes / No

Has your weight increased by more than 10 kg since you were 20 years old?

Yes / No

What is the condition of your teeth or mouth when eating?

· I can chew and eat anything.

· I have concerns about my teeth, gums, or bite, and I sometimes have difficulty chewing.

· I can hardly chew.

Have you participated in any of the following sports in the past?

- Baseball
- Tennis
- Basketball
- Swimming
- Long-distance running
- Track and field
- Soccer
- Volleyball
- Mountain climbing
- Other (please specify)

Are you currently engaging in any regular sports activities?

(You may select multiple answers)

- Brisk walking or continuous walking for more than 30 minutes
- Jogging
- Tennis
- Baseball
- Swimming
- Soccer
- Golf
- Aerobics
- Jazz dance

- Gym/fitness club
- Other

Do you perform light exercises that make you sweat for more than 30 minutes, at least 2 days a week, and have been doing so for over a year?

Yes / No

In your daily life, do you walk or engage in equivalent physical activities for more than 1 hour per day?

Yes / No

Compared to others of the same age and gender, do you walk faster?

Yes / No

Smoking and Drinking Habits

What is your current smoking status?

- I currently smoke.
- I used to smoke but quit.
- I have never smoked.

At what age did you start smoking?

(Write the age)

At what age did you quit smoking?

(If applicable, write the age)

For those who used to smoke: On average, how many cigarettes did you smoke per day?

- Not smoking
- 1 to 5 cigarettes
- 6 to 10 cigarettes
- 11 to 20 cigarettes
- 21 to 40 cigarettes
- More than 41 cigarettes

For current smokers: How many cigarettes do you currently smoke per day?

- Not smoking
- 1 to 5 cigarettes
- 6 to 10 cigarettes
- 11 to 20 cigarettes
- 21 to 40 cigarettes
- More than 41 cigarettes

How often do you drink alcohol?

(Every day / Occasionally / Rarely or never)

What type of alcohol do you drink?

(Sake, Beer, Whisky, etc. Select all that apply)

How much do you typically drink?

(Convert your alcohol consumption into the equivalent of 1 "gō" of sake, which is 180ml)

- Less than 1 "gō"
- 1 to 2 "gō"
- 2 to 3 "gō"
- More than 3 "gō"

Dietary Habits

How long do you usually wait between your evening meal and going to bed?

- Less than 1 hour
- 1 to 2 hours
- More than 3 hours

Do you eat quickly compared to others?

- Fast
- Normal
- Slow

Do you eat snacks or drink sweet beverages outside of your main meals?

- Every day
- Sometimes
- Rarely

How often do you eat salty foods (such as pickled foods, miso soup, etc.)?

- Every meal
- Once a day
- Rarely or never

(25) Do you have any of the following eating habits?

(You may select multiple answers)

- Eating dinner close to bedtime
- Eating dinner within 2 hours before going to bed at least 3 times a week
- Drinking alcohol and hardly eating any food

- Skipping breakfast at least 3 times a week
- Frequently drinking sugary soft drinks or canned coffee
- Frequently eating sweet foods
- Frequently eating fried foods or nuts
- Eating out more than twice a day
- Rarely eating seaweed or vegetables
- Rarely consuming milk, yogurt, or other dairy products
- Eating until you are full
- Frequently consuming animal fats

<u>For Women:</u>

Menstrual Cycle

Are you currently menstruating?

Yes / No

If no, have you already experienced menopause?

Yes / No

Age when menstruation started:

(Write age)

If applicable, age of menopause:

(Write age)

Pregnancy History

Have you ever been pregnant? How many times?

How many times have you given birth?

Breast Cancer Screening

Have you ever had a breast cancer screening?

What was the result?

Normal / Abnormal

<u>Medical History and Symptoms</u>

Do you have any of the following symptoms?

(If yes, check the corresponding box)

- Fatigue
- Loss of appetite
- Nausea

- Chest pain
- Palpitations
- Shortness of breath
- Coughing

Medical History

- Have you ever been diagnosed with a chronic disease?
- Have you had surgery in the past?
- Are you currently taking any medication?

Current Symptoms

Please check any symptoms that you have experienced in the past two months:

General Symptoms:

- I get tired easily.
- I've lost my appetite recently.
- I've lost weight recently.
- I've been sweating a lot, especially at night.
- I've had a fever recently.

Respiratory Symptoms:

- I've been coughing.
- I sometimes have shortness of breath.
- I've been coughing up phlegm.
- I sometimes feel pain in my chest.
- I sometimes feel tightness or discomfort in my chest.

Heart-related Symptoms:

- I've been told I have a heart murmur.
- I sometimes feel palpitations or irregular heartbeats.
- I've had chest pain during physical exertion.
- I feel short of breath or have discomfort in my chest when walking up a slope or stairs.

Digestive Symptoms:

- I often feel nauseous.
- I've noticed that my tongue looks coated (white or yellowish).
- I often feel that food gets stuck in my throat or chest.
- I experience heartburn.

- I feel a burning sensation in my stomach (pit of the stomach).
- I sometimes feel pain in my stomach.
- I often experience bloating, especially after eating.
- I've had constipation recently.
- I've had diarrhea recently.
- I've noticed blood in my stool.
- My stool color has been unusually dark or black.

Urinary Symptoms:

- I've had difficulty urinating.
- I feel the need to urinate urgently but can't always make it to the toilet.
- I've noticed that my urine stream is weak.
- I've had pain while urinating.
- I sometimes feel like my bladder isn't completely empty after urinating.
- I've experienced frequent urination, especially at night.

Neurological Symptoms:

- I've had dizziness or lightheadedness.
- I've had headaches recently.
- I've noticed that I feel shaky or unsteady.
- I sometimes see flashing lights or spots in my vision.
- I've had difficulty hearing recently.
- I've been experiencing ringing in my ears.

Joint and Muscle Symptoms:

- I sometimes feel pain or stiffness in my neck or shoulders.
- I've had lower back pain.
- I've experienced joint pain or swelling.
- I've had muscle cramps or stiffness.

<u>Allergies and Drug Reactions</u>

Do you have any allergies?

Yes / No (If yes, please specify the allergen: dust, food, medicine, etc.)

Have you ever had a bad reaction to a medical test (such as a blood test, barium swallow, mammogram, etc.)?

Yes / No (If yes, please provide details)

Have you ever had an adverse reaction to an injection (such as Buscopan or other drugs used during a test)?

Yes / No (If yes, please provide details)

Are you currently taking any blood-thinning medications (such as Warfarin, Aspirin, etc.)?

Yes / No

Are you taking any other medications?

Yes / No (If yes, please provide the name of the medication)

Digestive System Examination

How long has it been since your last meal?

(Please write the number of hours)

Have you had a barium X-ray exam in the past?

Yes / No (If yes, where did you have the exam?)

Have you had a gastroscopy (camera) exam?

Yes / No (If yes, where did you have the exam?)

Have you had a colonoscopy exam?

Yes / No (If yes, where did you have the exam?)

Have you ever been told that you have Helicobacter pylori bacteria in your stomach?

Yes / No (If yes, have you undergone treatment to eradicate it? Yes / No / Not sure)

Table S2. Comparison of Laboratory and Biochemical Parameters by OPLL Status

Item	Unit	Total	OPLL(-)	OPLL(+)	p-value
RBC	($\times 10^6$/μL)	4.73±0.48	4.73±0.48	4.73±0.49	0.933
WBC	($\times 10^3$/μL)	5.35±1.51	5.32±1.48	5.43±1.59	0.566
Hb	(g/dL)	14.41±1.53	14.44±1.60	14.40±1.51	0.778
Ht	(%)	42.90±4.08	42.88±4.02	42.94±4.25	0.925
Plt	($\times 10^3$/μL)	253.7±58.4	254.9±56.7	250.0±63.4	0.132
Na	(mmol/L)	140.1±1.6	140.1±1.5	140.2±1.6	0.66
K	(mmol/L)	4.23±0.32	4.23±0.31	4.21±0.33	0.208
Cl	(mmol/L)	103.7±2.0	103.7±1.9	103.5±2.1	0.172
Ca	(mg/dL)	9.33±0.34	9.33±0.34	9.32±0.35	0.918
TSH	(μIU/mL)	1.76±1.20	1.75±1.08	1.78±1.53	0.401
FT4	(ng/dL)	1.29±0.30	1.30±0.21	1.26±0.16	0.055
TP	(g/dL)	7.17±0.38	7.15±0.38	7.23±0.38	0.006*
Alb	(g/dL)	4.48±0.27	4.49±0.27	4.44±0.27	0.02*
T-Chol	(mg/dL)	211.0±35.3	209.0±34.7	217.2±36.5	0.029
HDL	(mg/dL)	60.50±15.44	60.52±15.67	60.42±14.69	0.81
LDL	(mg/dL)	121.1±31.7	119.4±31.3	126.5±32.2	0.021*
TG	(mg/dL)	123.2±129.6	120.6±129.5	131.1±129.6	0.116
AST	(U/L)	23.98±19.41	23.76±21.22	24.69±12.03	0.039
ALT	(U/L)	27.39±25.86	26.77±26.49	29.32±23.66	0.186
γ-GTP	(U/L)	44.93±55.53	46.07±60.28	41.35±36.56	0.494
T-Bil	(mg/dL)	0.94±0.33	0.93±0.32	0.96±0.35	0.296
LDH	(U/L)	172.8±30.2	171.9±30.2	175.6±30.1	0.1*
ALP	(U/L)	65.09±19.96	64.51±20.16	66.92±19.20	0.065
ChE	(U/L)	348.6±77.1	343.9±76.1	363.8±78.3	0.007*
HbA1c	(%)	5.68±0.66	5.62±0.53	5.88±0.94	<0.001*
BS	(mg/dL)	103.0±19.6	101.3±15.3	108.3±29.6	<0.001*
UA	(mg/dL)	5.58±1.50	5.58±1.51	5.58±1.44	0.864
Cre	(mg/dL)	0.79±0.16	0.80±0.16	0.78±0.17	0.093
eGFR	(mL/min/1.73m^2)	75.70±13.49	76.05±13.37	74.61±13.80	0.218
Fe	(μg/dL)	107.8±38.4	107.5±38.3	109.1±39.0	0.805
CRP	(mg/dL)	0.15±0.23	0.14±0.23	0.17±0.22	<0.001*

ESR at 60min.	(mm/h)	11.15±8.45	10.45±8.16	13.36±8.94	<0.001*
CEA	(ng/mL)	1.72±1.18	1.70±1.20	1.79±1.08	0.204
CA19-9	(U/mL)	10.12±6.83	9.51±5.88	12.22±9.11	<0.001*
AFP	(ng/mL)	3.48±2.08	3.49±2.10	3.45±2.01	0.788
CA125	(U/mL)	13.74±11.92	13.64±10.94	14.02±14.22	0.688
CYFRA21-1	(ng/mL)	1.70±0.85	1.69±0.84	1.72±0.85	0.535
SCC	(ng/mL)	1.56±1.76	1.54±1.44	1.62±2.55	0.048*
proGRP	(pg/mL)	43.47±12.49	43.11±11.38	44.37±14.88	0.923
NSE	(ng/mL)	12.63±3.26	12.68±3.35	12.46±2.90	0.619
fPSA	(ng/mL)	0.32±0.20	0.32±0.19	0.33±0.23	0.891
CA15-3	(U/mL)	9.32±4.72	8.85±4.12	10.59±5.86	0.747
SLX	(U/mL)	32.24±8.91	32.33±9.35	31.99±7.58	0.922
*indicates statistically significant					

Table S3. Comparison of Questionnaire Results on Lifestyle, Dietary Habits, and Physical Activity by OPLL Status

Sex									
		female	male	Total	p-value				
OPLL(-)	Count	409	601	1010	0.2				
	%	28.4%	41.7%	70.0%					
OPLL(+)	Count	191	241	432					
	%	13.2%	16.7%	30.0%					
Blood type									
		A	AB	B	O	n/a	Total	p-value	
OPLL(-)	Count	312	90	193	259	156	1010	0.752	
	%	21.6%	6.2%	13.4%	18.0%	10.8%	70.0%		
OPLL(+)	Count	129	31	87	120	65	432		
	%	8.9%	2.1%	6.0%	8.3%	4.5%	30.0%		
How intense are your daily life activities?									
		mostly seated	mainly seated for desk work	mostly standing	heavy labor	Total	p-value		
OPLL(-)	Count	282	341	87	34	744	0.143		
	%	26.8%	32.4%	8.3%	3.2%	70.8%			
OPLL(+)	Count	139	120	36	12	307			
	%	13.2%	11.4%	3.4%	1.1%	29.2%			
How many hours do you work per day?									
		0-3hrs	3-6hrs	6-9hrs	9-12hrs	12-15hrs	>15hrs	Total	p-value
OPLL(-)	Count	93	117	303	198	47	12	770	0.425
	%	8.6%	10.8%	28.1%	18.30%	4.0%	1.1%	71.3%	
OPLL(+)	Count	43	77	98	68	22	2	310	
	%	4.0%	7.1%	9.1%	6.3%	2.0%	0.2%	28.7%	
How many hours do you sleep?									
		0-5yrs	5-7hrs	8-9hrs	>9hrs	Total	p-value		
OPLL(-)	Count	79	682	237	7	1005	0.07		
	%	5.0%	47.5%	16.5%	0.5%	70.0%			
OPLL(+)	Count	47	293	84	6	430			
	%	3.3%	20.4%	5.9%	0.4%	30.0%			
Do you get enough rest from sleep?									

		No	Yes	Total	p-value				
OPLL(-)	Count	528	369	897	0.052				
	%	41.8%	29.2%	71.1%					
OPLL(+)	Count	193	172	365					
	%	15.3%	13.6%	28.9%					
Has your weight increased by 10 kg or more since you were 20 years old?									
		No	Yes	Total	p-value				
OPLL(-)	Count	484	413	897	0.081				
	%	38.4%	32.8%	71.2%					
OPLL(+)	Count	176	187	363					
	%	14.0%	14.8%	28.8%					
Sports previously participated in: Baseball									
		No	Yes	Total	p-value				
OPLL(-)	Count	391	142	533	0.639				
	%	53.2%	19.3%	72.5%					
OPLL(+)	Count	152	50	202					
	%	20.7%	6.8%	27.5%					
Sports previously participated in: Tennis									
		No	Yes	Total	p-value				
OPLL(-)	Count	370	138	508	1				
	%	52.2%	19.5%	71.7%					
OPLL(+)	Count	147	54	201					
	%	20.7%	7.6%	28.3%					
Sports previously participated in: Basketball									
		No	Yes	Total	p-value				
OPLL(-)	Count	398	109	507	0.032*				
	%	57.0%	15.6%	72.6%					
OPLL(+)	Count	164	27	191					
	%	23.5%	3.9%	27.4%					
Sports previously participated in: Swimming									
		No	Yes	Total	p-value				
OPLL(-)	Count	390	123	513	0.922				
	%	55.0%	17.3%	72.4%					
OPLL(+)	Count	148	48	196					
	%	20.9%	6.8%	27.6%					

Sports previously participated in: Long-distance running									
		No	Yes	Total	p-value				
OPLL(-)	Count	435	55	490	0.2				
	%	64.7%	8.2%	72.9%					
OPLL(+)	Count	168	14	182					
	%	25.0%	2.1%	27.1%					
Sports previously participated in: Soccer									
		No	Yes	Total	p-value				
OPLL(-)	Count	408	87	495	0.012*				
	%	60.0%	12.8%	72.8%					
OPLL(+)	Count	167	18	185					
	%	24.6%	2.6%	27.2%					
Sports previously participated in: Volleyball									
		No	Yes	Total	p-value				
OPLL(-)	Count	405	101	506	0.014*				
	%	58.4%	14.6%	72.9%					
OPLL(+)	Count	166	22	188					
	%	23.9%	3.2%	27.1%					
Sports previously participated in: Mountain climbing									
		No	Yes	Total	p-value				
OPLL(-)	Count	440	35	475	0.123				
	%	66.3%	5.3%	71.5%					
OPLL(+)	Count	168	21	189					
	%	25.3%	3.2%	28.5%					
Do you walk or perform an equivalent physical activity for at least 1 hour a day?									
		No	Yes	Total	p-value				
OPLL(-)	Count	536	362	898	0.705				
	%	42.2%	28.7%	71.1%					
OPLL(+)	Count	213	152	365					
	%	16.9%	12.0%	28.9%					
What is your current smoking status?									
		current smoker	smoking history	never	Total	p-value			
OPLL(-)	Count	225	389	396	1010	0.042*			
	%	15.6%	27.0%	27.5%	70.0%				

OPLL(+)	Count	71	180	181	432				
	%	4.9%	12.5%	12.6%	30.0%				
How often do you drink alcohol?									
		daily	occasionally	none	Total	p-value			
OPLL(-)	Count	319	303	386	1008	0.047*			
	%	22.2%	21.0%	26.8%	70.0%				
OPLL(+)	Count	117	120	195	432				
	%	8.1%	8.3%	13.5%	30.0%				
How much time do you have between dinner and bedtime?									
		0hrs	1-2hrs	2-3hrs	>3hors	Total	p-value		
OPLL(-)	Count	28	177	273	325	803	0.563		
	%	2.5%	15.7%	24.20%	28.8%	71.2%			
OPLL(+)	Count	7	66	112	140	325			
	%	0.6%	5.9%	9.9%	12.4%	28.8%			
Do you eat faster than others?									
		Yes	Same	No		p-value			
OPLL(-)	Count	359	444	78	881	0.936			
	%	29.0%	35.9%	6.3%	71.2%				
OPLL(+)	Count	142	184	31	357				
	%	11.5%	14.9%	2.5%	28.8%				
Do you consume snacks or sweet beverages outside of your 3 main meals?									
		daily	occasionally	never	Total	p-value			
OPLL(-)	Count	228	447	212	887	0.446			
	%	18.3%	35.8%	17.0%	71.1%				
OPLL(+)	Count	90	195	76	361				
	%	7.2%	15.6%	6.1%	28.9%				
How often do you eat salty foods?									
		Every time	1-2times	once	None	Total	p-value		
OPLL(-)	Count	196	143	377	76	792	0.278		
	%	17.5%	12.8%	33.%	6.80%	70.7%			
OPLL(+)	Count	66	66	158	39	329			
	%	5.9%	5.9%	14.1%	3.5%	29.3%			
Eating habits: Having dinner close to bedtime									
		No	Yes	Total	p-value				
OPLL(-)	Count	379	129	508	0.278				

	%	54.1%	18.4%	72.5%					
OPLL(+)	Count	156	37	193					
	%	22.3%	5.3%	27.5%					
Eating habits: Frequently eating sweets									
		No	Yes	Total	p-value				
OPLL(-)	Count	300	261	561	0.874				
	%	38.3%	33.3%	71.6%					
OPLL(+)	Count	117	105	222					
	%	14.9%	13.4%	28.4%					
Eating habits: Often eating out for 2 or more meals a day									
		No	Yes	Total	p-value				
OPLL(-)	Count	400	93	493	0.586				
	%	58.0%	13.5%	71.4%					
OPLL(+)	Count	164	33	197					
	%	23.8%	4.8%	28.6%					
Eating habits: Rarely consuming dairy products such as milk or yogurt									
		No	Yes	Total	p-value				
OPLL(-)	Count	357	164	521	0.278				
	%	49.0%	22.5%	71.6%					
OPLL(+)	Count	154	53	207					
	%	21.2%	7.3%	28.4%					
Eating habits: Frequently consuming animal fat									
		No	Yes	Total	p-value				
OPLL(-)	Count	331	222	553	0.871				
	%	42.8%	28.7%	71.4%					
OPLL(+)	Count	134	87	221					
	%	17.3%	11.2%	28.60%					
Do you have swelling or pain in your wrists or finger joints?									
		No	Yes	Total	p-value				
OPLL(-)	Count	425	88	513	0.001*				
	%	58.9%	12.2%	71.1%					
OPLL(+)	Count	148	61	209					
	%	20.5%	8.4%	28.9%					
*Indicates statistically significant									

Table S4. Association Between Dyslipidemia and OPLL Status in an Asymptomatic Health Screening Population

Dyslipidemia					
		No	Yes	Total	p-value
OPLL(-)	Count	589	421	1010	0.603
	%	40.8%	29.2%	70.0%	
OPLL(+)	Count	259	173	432	
	%	18.0%	12.0%	30.0%	
